# ID 329 - Realização do Exame de Eletrocardiograma por Equipes de Saúde da Família no Brasil: um estudo descritivo em 10 anos

**DOI:** 10.5327/2237-9622.2025.v34s1.329

**Published:** 2025-11-25

**Authors:** Aline Gonçalves Pereira, Thaís Barbosa de Oliveira, Rafaela de Paula Sales

**Keywords:** Atenção Primária à Saúde, eletrocardiograma, equidade no acesso aos serviços de saúde

## Abstract

**Introdução:**

O eletrocardiograma (ECG) é um exame diagnóstico fundamental para a detecção precoce de doenças cardiovasculares, uma das principais causas de mortalidade no Brasil e no mundo. Na Atenção Primária à Saúde (APS), as equipes de Saúde da Família (eSF) desempenham um papel essencial na prevenção, no diagnóstico e no acompanhamento de diversas condições de saúde, incluindo as cardiovasculares. Com a descentralização dos serviços de saúde e a ampliação do acesso, houve um esforço para que a oferta de exames como o ECG fosse integrada às práticas dessas equipes. No entanto, ainda há lacunas no entendimento sobre a distribuição, a periodicidade e o impacto da realização desses exames no território nacional. Este estudo objetivou descrever a realização de ECG por eSF no Brasil ao longo de um período de dez anos, buscando fornecer uma visão panorâmica das desigualdades regionais e das tendências temporais na oferta dessa tecnologia.

**Método:**

Trata-se de um estudo quantitativo descritivo sobre a realização de ECG por eSF entre os anos de 2014 e 2023 no Brasil. Os dados são de fonte secundária, extraídos da plataforma pública do Sistema de Informação em Saúde para a Atenção Básica (Sisab). No Sisab, consultou-se o total de ECG conforme o código 0211020036 registrado no Sistema de Gerenciamento da Tabela de Procedimentos, Medicamentos e Órteses, Próteses e Materiais Especiais (OPM) do Sistema Único de Saúde (SUS).

**Resultados:**

Entre os anos de 2014 e 2023, foram realizados 1.557.486 ECG por eSF no País. O ano de 2019 registrou o maior número de atividades (15,20%, n=236.804) em comparação aos outros anos. Quando se estratifica a realização desse procedimento por Grande Região, observou-se uma predominância no Sudeste (53,56%, n=876.594), seguido pelo Sul (20,43%, n=334.114). Em contrapartida, as Grandes Regiões Nordeste (12,36%, n=202.312), Centro-Oeste (5,23%, n=85.576) e Norte (3,52%, n=57.599) apresentaram os menores registros. Sob uma perspectiva histórica, no período de 2019 a 2023, todas as Grandes Regiões apresentaram declínio de realização de ECG, com uma variação percentual de -57,34% no Centro-Oeste; -45,00% no Nordeste, -40,66% no Sudeste; -24,50% no Sul; e -1,97% no Norte.

**Conclusão:**

O estudo evidencia uma acentuada desigualdade regional na realização de ECG pelas eSF no Brasil, com destaque para o Sudeste, que concentrou mais da metade dos exames, possivelmente em razão de melhores recursos estruturais e financeiros. As Regiões Norte e Centro-Oeste, por outro lado, mostraram os menores índices, apontando para a necessidade de maior investimento e acesso a esses exames em áreas com desafios logísticos e geográficos. A partir de 2019, todas as regiões registraram uma queda na realização de ECG, com destaque negativo para o Centro-Oeste e o Nordeste, provavelmente influenciada por fatores como mudanças nas políticas de saúde, menor investimento na APS e os efeitos da pandemia de covid-19. Apesar da expansão inicial do serviço, as disparidades regionais permanecem, exigindo ações coordenadas para garantir uma distribuição mais equitativa e o fortalecimento das eSF, sobretudo nas áreas mais vulneráveis, na adesão da tecnologia e na oferta de exames.

